# Adaptive molecular evolution of *MC1R* gene reveals the evidence for positive diversifying selection in indigenous goat populations

**DOI:** 10.1002/ece3.2919

**Published:** 2017-06-07

**Authors:** Hafiz Ishfaq Ahmad, Guiqiong Liu, Xunping Jiang, Chenhui Liu, Yuqing Chong, Huang Huarong

**Affiliations:** ^1^ Key Laboratory of Agricultural Animal Genetics, Breeding and Reproduction of the Ministry of Education College of Animal Science and Technology Huazhong Agricultural University Wuhan China

**Keywords:** goat, maximum likelihood, positive selection, SNP

## Abstract

Detecting signatures of selection can provide a new insight into the mechanism of contemporary breeding and artificial selection and further reveal the causal genes associated to the phenotypic variation. However, the signatures of selection on genes entailing for profitable traits between Chinese commercial and indigenous goats have been poorly interpreted. We noticed footprints of positive selection at *MC1R* gene containing SNPs genotyped in five Chinese native goat breeds. An experimental distribution of *F*_ST_ was built based on approximations of *F*_ST_ for each SNP across five breeds. We identified selection using the high *F*_ST_ outlier method and found that *MC1R* candidate gene show evidence of positive selection. Furthermore, adaptive selection pressure on specific codons was determined using different codon based on maximum‐likelihood methods; signature of positive selection in mammalian *MC1R* was explored in individual codons. Evolutionary analyses were inferred under maximum likelihood models, the HyPhy package implemented in the DATAMONKEY Web Server. The results of codon selection displayed positive diversifying selection at the sites were mainly involved in development of genetic variations in coat color in various mammalian species. Positive diversifying selection inferred with recent evolutionary changes in domesticated goat *MC1R* provides new insights that the gene evolution may have been modulated by domestication events in goats.

## 1 Introduction

Coat color variation in mammals is one of the most distinctive phenotypic traits, which put a significant biological and economic impact, and it can be enlightened by various selective pressures including communication, concealment, and regulation of physiological processes (Andersson, 2001; Chen et al., 2009). The genetic basis of coat color legacy has been unrevealed in farm animals including goat, sheep, and cattle (Bemji et al., 2012; Fontanesi et al., 2009; Xi et al., 2012). The major genetic determinant of melanin content in mammals is melanocortin receptor (*MC1R*) gene (Kerje, Lind, Schütz, Jensen, & Andersson, 2003; Lin & Fisher, 2007), which is seven transmembrane G‐protein‐coupled receptor, expressed on melanocyte surface (Javanmard, Arafnajad, Arpanahi, & Moradi, 2015), and it is affecting the production regulation of two pigments; eumelanin and pheomelanin (Liu et al., 2016; Oetting, Austin, & Bennett, 2009). The function of *MC1R* determines the pigmentation as increased *MC1R* activity enhances the eumelanin (black/dark, brown pigment) production, whereas decreased *MC1R* activity enhances pheomelanin (red/yellow pigment) production (Barsh, 1996; Robbins et al., 1993). *MC1R* is a single exon gene included 954 bp complete coding sequences with a part of 5′‐ and 3′‐ untranslated regions and has seven transmembrane domains with standard 317 amino acid length (Fontanesi et al., 2009; Shimada, Sato, Aplin, & Suzuki, 2009). *MC1R* gene mutations associated with color phenotype have extensively been studied in different livestock species including goat (Fontanesi et al., 2009), sheep (Våge et al., 2003), and cattle (Klungland, Vage, Gomez‐Raya, Adalsteinsson, & Lien, 1995). In goats, a wide variation in coat color has been accounted by agouti loci in several breeds (Robbins et al., 1993). The persistence of dominant black allele and a recessive red allele has been documented in few breeds (Sponenberg, 1990), and on the other hand, wild‐type allele of agouti made different phenotypic effects in other species, as in Boer goat the missense mutation (p.k226E) in *MC1R* gene was associated with red color phenotype (Wu et al., 2006). The *MC1R* has primary role in coat color evolution; it may be possible to elucidate the long‐term coat color evolutionary propensity in a specific ancestry by evaluating the amino acid substitution rate in coding region across the taxa over evolutionary time (Nadeau, Burke, & Mundy, 2007). The identification of genes undergone positive selection has vital role to understand the livestock adaptation to various environmental changes to reveal insights on the selection history. The adaptive evolution was measured by synonymous and nonsynonymous substitution (dN/dS) ratio, as in lion tamarin and mouse, the *MC1R* sequences have evolved with amino acid changes, which have higher ratio of dN/dS reflecting specific evolutionary episode related coat color (Mundy & Kelly, 2003). Acquisition of new functions in genes is credited to adaptive selection pressures in close association with phenotypes and fitness of organisms (Wright & Rausher, 2010). Adaptive selective pressure on genes has also been reported to be indicators of functional adaptations developed during the evolution of species that has the tendency of promoting species functional diversification (Vamathevan et al., 2008). Color variations within species present an opportunity to understand the genetic system underlying phenotypic changes and evolutionary meaning of phenotypic characteristics (Hoekstra, Drumm, & Nachman, 2004), as well as coat color provides precious evidence to the natural history of the decree of taxonomic issues for subspecies discrimination (Nachman, Hoekstra, & D'Agostino, 2003). Understanding the evolutionary footprint of *MC1R* gene will therefore provide valuable information for reconstructing evolutionary history of species and other functional genes, and may provide useful insights into the design of marker‐assisted selection and breeding for genetic improvement in goats. In this study, we investigated the molecular evolutionary signatures that may exert selection processes in the *MC1R* gene in goats and identified evolution footprints that may influence adaptation to different environments.

## 2 Materials and Methods

### 2.1 Ethics statement

All the essential experimental protocols included in this study were validated by the Law of Animal Husbandry in People's Republic of China (Dec 29, 2005). The entire protocols for sample collection were reviewed and legalized by the Biological Studies, Animal Care and Use Committee of National Animal Husbandry Services, Hubei, China. All measures were adopted to diminish discomfort of the animals during sample collection.

### 2.2 Animals selection

A total of 526 individuals present in different regions, including Macheng Hubei black‐boned goat from Jinyang farm (*n* = 167), Tonshan Hubei black‐boned goat from Tongshan black‐boned goat corporation (*n* = 107), Youzhou black goat from Youzhou black goat farm (*n* = 138), Nanjing yellow goat (*n* = 84), and Hubei Boer goat from householders (*n* = 30) were selected for this study. All goats included in this study were reared under the traditional system and were checked in order to identify domestic variants according to coat color. The selected animals were unrelated and were from different geographical regions to maximize the genetic variation among individuals. The sampling was carried out in such a way that maximized spatially unrelated individuals to ensure the breed representativeness and to maximize the genetic diversity among individuals. The genomic DNA samples of 526 individuals were pooled together for selection analysis.

### 2.3 Sample collection and DNA extraction

Genomic DNA was extracted from blood samples using the TIAGEN DNA Mini Kit (TIAGEN Bio, China). A Nanodrop 2000 (Thermo Scientific, USA) was used to quantify DNA concentration and then stored at −20°C for genotyping.

### 2.4 Genotyping and polymorphism

The genomic DNA was used to study 13 gene extensions to find out the possible loci in all genomic samples. PCR was carried out on a PCR thermo cycler (Bio‐Rad Life Science research) in a total volume of 20 μl containing 12.5 μl PCR master mix (Takara Clontech, Japan), 1.6 μl of both forward and reverse primers, 0.5 μl DNA template, and 5.4 μl nuclease free water. The PCR profile was 35 cycles of 30 s at 95°C, 40 s at 65°C, 40 s at 72°C, 10 min at 72°C and was held at 4°C. The PCR product was analyzed using 2% agarose gel electrophoresis. The PCR products were sequenced using ABI 3100 automated sequencer (Applied Biosystem, Foster city, CA, USA). The primers used in this study are provided in Table S2 (Capra et al., 2017).

### 2.5 Positive selection analysis

To detect loci under selection, Beaumont and Nichols's approach (Narum & Hess, 2011) implemented in LOSITAN was used. Fixation index (*F*_ST_) and *p*‐values for each locus were calculated using allele frequencies based on heterozygosity simulations, and predictable *F*_ST_ value was 0.102 in 526 individuals of five populations, and 13 loci. This method presented the contrary selection by finding the outlier with *F*_ST_ values more than probable overcoming for heterozygosity (Huber, DeGiorgio, Hellmann, & Nielsen, 2016). To build up the population data set, 100,000 simulations were utilized in real data. Quantiles were assumed for transitional joint allocation of *F*_ST_ against mean heterozygosity with a 95% confidence limit. The outlier was predicted by the loci expressing outside the simulated neutral distribution with differentiating behavior (Antao, Lopes, Lopes, Beja‐Pereira, & Luikart, 2008). Similarity in *F*_ST_ /H_E_ values for all loci indicates a shared demographic history. Loci showing large amounts of differentiation may mark the regions of genome that have been subjected to directional selection, while loci showing a small amount of differentiation may mark the region of the genome subjected to balance the selection (Holsinger & Weir, 2009). The Nucleotide and amino acid sequences of *MC1R* gene were retrieved from GenBank (www.ncbi.nlm.nih.gov/genbank), Ensembl (http://useast.ensembl.org/index.html), and UniProt (http://www.uniprot.org), and accomplished sequences of proteins were aligned through using ClustalW, as implemented in MEGA6.0 program (Tamura, Stecher, Peterson, Filipski, & Kumar, 2013). The phylogenetic tree of mammalian *MC1R* gene was constructed with MEGA 6.0 software package based on maximum likelihood. Evidence of positive selection of *MC1R* gene was inferred by estimating the rates of synonymous and nonsynonymous changes at each site in aligned sequence through various approaches such as single likelihood ancestor counting (SLAC), fixed effect likelihood (FEL) method, and internal fixed effect likelihood (IFEL) methods (Pond & Frost, 2005; Pond & Muse, 2005; Pond et al., 2006). *p*‐values <.05 were considered as significant.

### 2.6 Evolutionary analysis of diversifying selection

Evolutionary analysis of diversifying selection was performed by various approaches to detect the episodic diversifying detection affecting individual codon sites. Mixed‐effects model evolution (MEME) combines the fixed effects to identify instances of both episodic diversifying selection and pervasive positive selection at the individual branch site level and fast unconstrained Bayesian approximation (FUBAR) using Markov Chain Monte Carlo (MCMC) routine, which ensures the robustness against model misspecification over predefined sites through approximate Bayesian method (Murrell et al., 2012; Pond et al., 2011). The fitting of MEME to alignment, MG94xREV codon model, was applied using parameter estimates ω = β/α fitted to the data using the GTR nucleotide model as initial values. The selective pressure was measured with two parameters β: β^−^ ≤ α and β^+^ (Pond & Frost, 2005; Yang, 2000), and the alternative model includes four parameters for each site: β^−^, β^+^, q^−^ and α estimating site to site substitution variability rates (Pond & Muse, 2005a). The values of *p* < .05 were considered as significant from the LRT based on χ^2^ asymptotic distribution (Pond & Frost, 2005).

## 3 Results

The sequence picture among five goat populations of various breeds revealed thirteen single nucleotide polymorphic sites with an average density 566 bases for each. These single nucleotide polymorphic sites were the consequence of outlier determination. Fixation selection Index‐based methods recognized *MC1R* loci as under selection prance in investigating goat breeds.

### 3.1 Positive selection of *MC1R* gene by FDIST analysis

**Figure 1:**
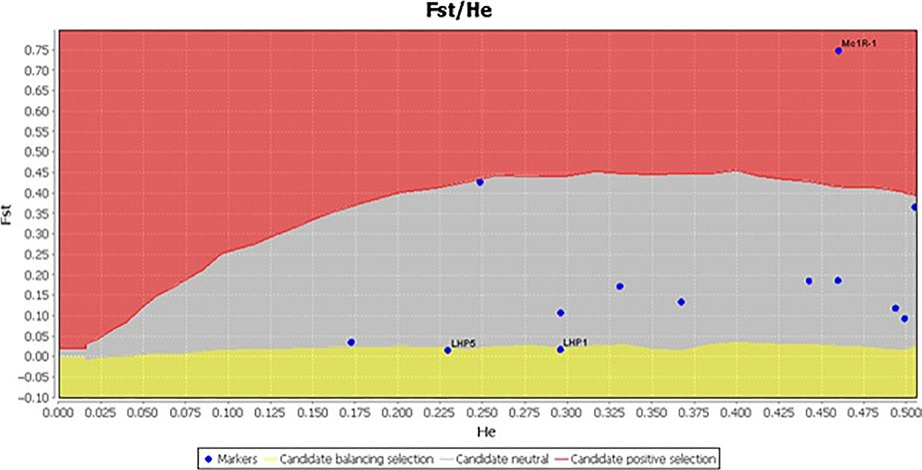
Candidate locus agouti under positive selection keeping the 95% confidence interval. *F*_ST_, fixation index; H_E_, Heterozygosity

The high *F*_ST_ outlier method corresponding to *F*_ST_ distribution was used to identify the loci subjected to positive selection. According to the empirical distribution of *F*_ST_ estimates, we selected the SNPs having high *F*_ST_ outlier corresponded to the upper 1% of the distribution as the loci under selection. In all five goat populations, 13 SNPs were determined from 13 genes to be subjected to natural or artificial selection. These loci were analyzed for positive selection, and the most locus undergone positive selection was *MC1R*1. Positive selection of *MC1R* gene was analyzed with LOSITAN FDIST analysis using outlier approach, *MC1R* gene showed a significant positive selection in the outlier area as there was a strong evidence of positive selection for this locus because of its low heterozygosity (Table 1 and Figure 1).

**Table 1:** Heterozygosity (He) and Fixation Index *F_ST_* values for each of 13 genotyped SNPs calculated by LOSITAN in five goat populations

SNP	Locus	Heterozygosity (*H*_E_)	Fixation index (*F*_ST_)	P*
***MC1R‐1***	Melanocortin receptor	**0.45981**	**0.74821**	0.99999*
*MC1R‐2*	Melanocortin receptor	0.50487	0.36586	0.94737
*Agouti‐1*	Agouti protein	0.49878	0.09401	0.27772
*LHP1*	Leutinizing hormone	0.29569	0.01821	0.01939
*LHP5*	Leutinizing hormone	0.22927	0.01597	0.00865
*GnRHR*	Gonadotrpinreleasing hormone	0.17240	0.03570	0.07813
*PGR1*	Prostaglandin receptor	0.49341	0.11916	0.37981
*FSHb*	Follicle stimulating hormone	0.24826	0.42750	0.96298
*Myf‐5*	Mayogenic factor	0.33079	0.17224	0.59913
*INHA*	Inhibin	0.44237	0.18577	0.58081
*NPY‐R1*	Neuropeptide Y	0.29587	0.10812	0.37418
*IGF‐1*	Insulin like growth factor	0.45943	0.18686	0.58423
*AA‐NAT*	Aralkylamine N‐acetyltransferase	0.36705	0.13483	0.43468

Bold value showing loci with high *F*_ST_ value. Significant: p* (Simulated *F*_ST_ < sample *F*_ST_).

Evolutionary evidence of positive selection was determined by estimating global ω values using single likelihood ancestor counting (SLAC) analysis, fixed effect likelihood (FEL), and internal fixed effect likelihood (IFEL) methods. The results showed that there was strong evidence of evolutionary positive selection of *MC1R* gene in mammals. The REL detected five sites under positive selection, and these sites were at 59, 218, 248, 327, and 364 positions; the IFEL detected three sites under positive selection at sites 59, 279, and 327 codons, while SLAC analysis and FEL analysis detected two sites under positive selection at positions 59, 279 and 59, 364, respectively, as narrated in Table 2. The position 59 was detected under positive selection in all four analyses, while IFEL analysis and SLAC analysis detected positive selection at the same sites. REL detected more positive sites than other analyses at 95% confidence interval. REL analysis was based on Bayes factor, and the values>20 were detected under positive selection. *p* values <.05 were considered as significant for other analysis, and all detected sites were significantly different and having *p*‐values<.05 (Table 2).

**Table 2:** Sites found under positive selection at *MC1R* gene

REL			IFEL			SLAC			FEL		
Positive selection sites	dN‐dS	Bayes Factor	Positive selection sites	dN‐dS	*p*	Positive selection sites	dN‐dS	*p*	Positive selection sites	dN‐dS	*p*
59	0.24	40.18	59	0.27	.03	59	2.385	.02	59	0.26	.02
218	0.11	41.56	279	0.76	.04	279	2.554	.04	364	0.19	.02
248	0.04	21.95	327	0.28	.04						
327	0.05	31.74									
364	0.15	255.7									

REL, IFEL, SLAC, and FEL test significance levels are given as *p*‐values, while empirical Bayes factors are given for REL. Significant values (*p* < .05), Bayes factors >20.

### 3.2 Evolutionary analysis of diversifying selection

The mixed‐effect model evolution analysis detected six sites undergone episodic diversifying selection. Among these sites, 59, 86, 108, 153, 179, and 364 were detected under episodic diversifying having *p* values<.05 (Table 3). This model also estimates the synonymous (α) and nonsynonymous (β) substitution rates, and the sites having values β > α were considered as significant and determined these sites under diversifying selection. The sites 59 was inferred to have experienced pervasive nonsynonymous substitution throughout the evolutionary history with *p* values .015, and this site evolved with β^+^ > α, as it is under positive selection in all analyses providing the strong evidence of positive selection of *MC1R* gene, whereas all other sites were conserved. The value of q was determined using Simes’ method to reduce the false discovery rate under strict neutral null model (Table 3). At codons 86, 108, 153, and 179, MEME obtained maximum‐likelihood estimate β^+^>α in other analyses, but failed to infer positive selection as the signal was nonsignificant.

**Table 3:** Mixed‐effect model evolution based episodic diversifying selection at *MC1R* gene

Codon	α	β^−^	Pr[β = β^−^]	β^+^	Pr[β = β^+^]	*p*‐value	q‐value
59	0	0	0.616	1.809	0.384	.015	1
86	0.722	0	0.954	103.8	0.046	.031	1
108	0.148	0	0.987	613.7	0.013	.005	1
153	2.186	0.502	0.958	38.36	0.042	.045	1
179	0.254	0	0.984	16.28	0.016	.021	1
364	0	0	1e‐09	0.454	1	.03	1

Table reports the distribution of synonymous (α) and nonsynonymous (β) substitution rates over sites inferred by MEME model where the branch proportion with β > α significantly >0. *p*‐values determined by χ^2^ distribution and q‐values obtained using Simes’ procedure to reduce false discovery rates under strict null neutral model.

**Figure 2:**
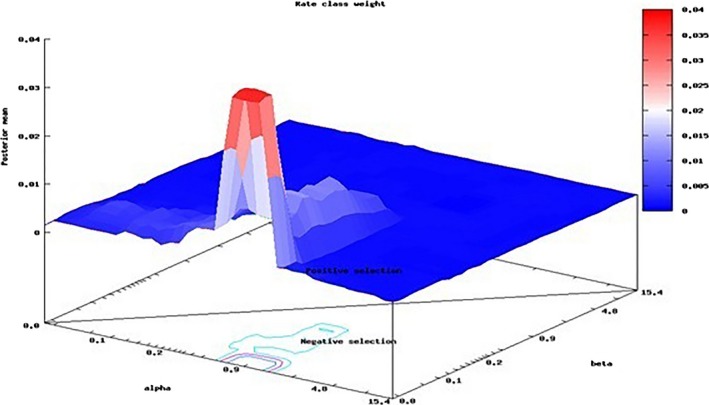
Fast unconstrained Bayesian approximation inferring pervasive diversifying selection based on synonymous (α) and nonsynonymous rates (β) substitution rate with continuous model parameters that vary from one site to another, illustrated in posterior alignment‐wide distribution of substitution rates Mean values. β = 0.76, β−α = −0.24, ω = 5.49 Weights. Pr[α > β] = 0.708, Pr[α = β] = 0.042, Pr[α < β] = 0.250

We used fast unconstrained Bayesian approximation using Markov Chain Monte Carlo approach (Murrell et al., 2013) to integrate over the uncertainty in posterior gene‐specific and site‐specific distributions identified three cites at *MC1R* gene under pervasive diversifying selection. Using the random effect models, employing the rate distribution with a large number of parameters detected evolutionary evidence of diversifying detection at 59, 279, and 364 positions at 95% confidence interval, and the values Pr[β>α] were considered as significant (Table 4). Using bivariate general discrete distribution, the selection was inferred by a class rate weight at each site undergone positive selection. The graph was plotted based on synonymous (α) and nonsynonymous (β) substitution rates and their posterior probability values with class rate weight at each site (Figure 2). The posterior mean value at site 59 is 0.927, for 279 is 0.924, and for 364 is 0.95, and the potential scale reduction factor values closer to one are indicative of MCMC convergence. The empirical Bayesian factor values for each site undergone positive selection were estimated with net effective sample size (Table 4).

**Table 4:** Fast unconstrained Bayesian approximation inferring pervasive diversifying selection at *MC1R* gene

Codon	α	β	β−α	Pr[β > α]	E.B. Factor	PSRF	Neff
59	0.08	0.26	0.18	0.927	38.32	0.998	1,892.62
279	0.22	0.67	0.45	0.924	36.66	0.998	1,560.56
364	0.04	0.21	0.16	0.95	56.49	1.004	591.746

α, The mean of posterior distribution with respect to data set wide distribution of rates of the empirical Bayes estimate of synonymous substitution rates; β, Posterior mean of nonsynonymous substitution rate; Pr [β > α], Posterior mean of the site level probability of positive selection; PSRF, Potential scale reduction factor and the values close to indicate MCMC convergence; Neff, The effective sample size from pooled chain.

### 3.3 Evolutionary finger printing of *MC1R* gene

**Figure 3:**
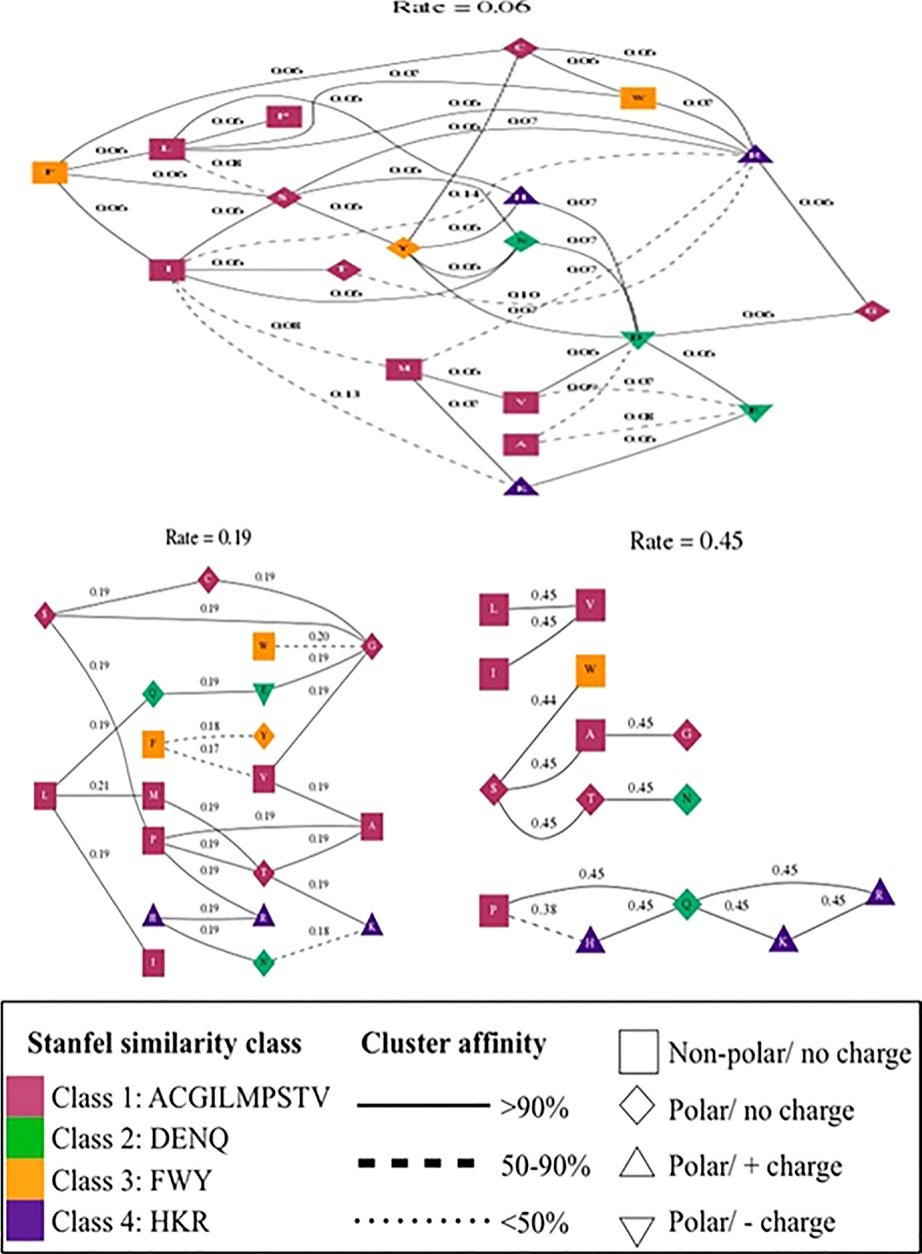
Evolutionary rate cluster in structured genetic algorithm models (GA) inferred from *MC1R* gene alignment. Each cluster labeled with maximum‐likelihood estimate of its rate inferred under genetic algorithm. The nodes (residues) are annotated by their biochemical properties and Stanfel class, and the rates (edges) are labeled with model averaged rate estimates

Evolutionary finger printing of *MC1R* gene was inferred through codon model evolution based on synonymous and nonsynonymous substitution rates using genetic algorithm. The codon model evolution (Delport et al., 2010; Pond, Scheffler, Gravenor, Poon, & Frost, 2010) used phylogenetic Markov model, including substitution rates, character frequencies (Pond, Delport, Muse, & Scheffler, 2010), clustering of amino acid substitution rates (Stanfel, 1996), and branch lengths through maximum‐likelihood estimation method. The codon model selection used 10,519 models based on likelihood log (log L) and modified Bayesian Information Criterion (mBIC). The best model was identified with log (L) = −12,600.3, mBIC = 27,114.7 has three (0.06, 0.19, 0.45) class rates with single rate dN/dS substitution estimates of 0.06 over 39 residue pair (Table 5), and these three class rates were further analyzed by genetic algorithm multirate models to evaluate the amino acid substitution rates during evolution. The genetic algorithm models identified the exchange rates at each class rate by estimating the nonsynonymous substitution rates residues labeled by Stanfel class (Figure 3; Stanfel, 1996). The evolutionary rate clusters, indicating the amino acid substitution rates at three class rates, and the substitution pair ACGILMPSTV have 90% substitution, whereas DENQ has 50% substitution, and FWY and HKR have substitution rates <50%.

**Table 5:** Codon model selection based on Modified Bayesian Information Criterion (mBIC)

*N*	Models	Credible	mBIC	ΔmBIC	dN/dS
1	1	0	27,594.2		0.16/75
2	3,302	0	27,204.6	389.6	0.07/45
3	6,680	1,948	27,114.7	89.9	0.06/39
4	536	1	27,121.3	−6.66	0.05/20

*N*, Number of rate classes included in models; Models, Genetic algorithm models; Credible, All the models evaluated by genetic algorithm within 9.21 mBIC unit (best model has credible values 0.01 or >1); mBIC, Modified Bayesian Information Criterion; ΔmBIC, mBIC for *N* rate classes compared to *N*−1 rate classes; dN/dS, Maximum‐likelihood estimates for each rate class.

The genetic relationship of *MC1R* gene of goat populations was illustrated by phylogenetic analysis of goat *MC1R* gene with other mammalian species. The analysis was performed using MEGA 6.0 program. The analysis was carried out to find out the relationship of goat MC1R with other animal species on the bases of genetic variations among various individuals, which has occurred during domestication events under the influence of selection pressure. Because domestication necessarily involves the separation of animals from their natural environment, the alterations in coat color during animal domestication could have been the result of a relaxation of the selection pressure against noncamouflaged coat. There were total 612 positions in the final data set, and the tree was constructed based on maximum likelihood by applying Neighbor joining and BioNJ algorithms. Based on open reading frame of *MC1R,* evolutionary kinship in the phylogenetic tree (Figure S1) indicated that goat (*C. hircus*) was the most closely related to sheep and cattle.

**Figure 4:**
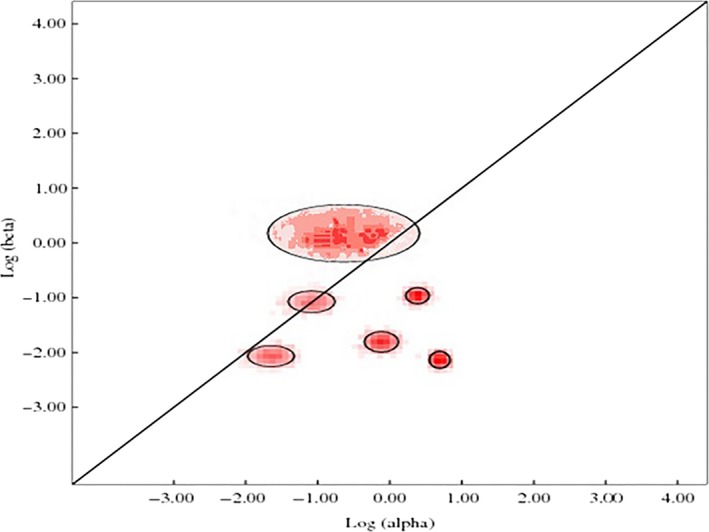
Evolutionary fingerprints of *MC1R* gene inferred from alignments on log scale, with the diagonal line corresponding the values α = β for neutral evolution. Dots in the circle indicating the ratio β/α and the area of circle represent the weight of rate classes. The points above the diagonal correspond positive selection and below the diagonal negative selection

The probability of site to site distribution of synonymous and nonsynonymous substitution rates exploited the ratio ω = β/α, which is estimated on the bases of likelihood log and Akaike information criterion (AIC) using five rate classes identified neutral evolution at *MC1R* gene. The likelihood log was −12,446.59833 for five class rates using 37 parameters, and AIC value was AIC: 24,967.19666. The nonsynonymous to synonymous ratio ω = β/α values for all five classes were 0.006, 0.178, 0.156, 0.386, and 0.831, respectively. The graph showing the neutral evolution and only a few sites under the circle above diagonal has positive evolution at the *MC1R* gene (Figure 4).

## 4 Discussion

The current advances in the vital index of genetic differences have projected narrative suggestions for exploration of the positive selection goals, which eventually would be supportive to enlighten the genetic drifts and selection roles in evolutionary mechanisms. Moreover, positive selection signature impedes the genomic regions that play significant roles. Consequently, exploring such regions will provide considerable support for identification of genetic deviations, which would facilitate the distraction of these functional regions and progression in phenotypic assortments. The illuminations of the genetic bases of different traits in most species have been studied by candidate gene approach. The identification of these candidate genes plays an important role in phenotypic variation in livestock population and provides new advancement in the evolutionary process and positive selection (Brown et al., 2013; Storz, Payseur, & Nachman, 2004).

The study of melanin pigmentation in vertebrates has led to the reporting of important target genes that may cause phenotypic variations and divergences in natural populations (Ducrest, Keller, & Roulin, 2008; Hoekstra & Coyne, 2007). One persistent result evolving from most studies is the contribution of the *MC1R* coding region in explanation of melanism variation, sometimes displaying shared mutations due to convergent evolution between coldly related species (Rompler et al., 2006). *MC1R* has been shown to play a significant role in various mechanisms such as sexual selection (Mundy et al., 2004), crypsis (Mullen & Hoekstra, 2008), and possibly immunity (Gangoso et al., 2011) although it is generally considered to have few pleiotropic effects (Ducrest et al., 2008). However, in our study, we found molecular evolution in *MC1R* coding sequences, as would be predictable as they were associated with positively selected regions in nearby locations. Our results are instead consistent with those obtained by Cheviron, Hackett, & Brumfield, 2006). In addition to *MC1R*, Agouti gene also interacts with *MC3R* and *MC4R* and has pleiotropic effects on energy expenditure, food intake (Ducrest et al., 2008).

Among the selection detection approaches, *F*_ST_ outlier approaches are widely used as *F*_ST_ outlier tests recognize the genes that have undergone divergent selection discriminating the large values of *F*_ST_ than expected values, and outlier approaches have been used in genomewide scans for determining selection signatures of genes potentially under the selection in the extreme tail of empirical distribution (Akey, Zhang, Zhang, Jin, & Shriver, 2002; Voight, Kudaravalli, Wen, & Pritchard, 2006; Wang, Kodama, Baldi, & Moyzis, 2006). *F*_ST_ outlier detected *MC1R*1 loci at *MC1R* gene under positive selection through estimation the relationship between *F*_ST_ and He (expected heterozygosity) and described the expected Wright's inbreeding coefficient *F*_ST_ distribution and this distribution identified *MC1R* as an candidate outlier loci subjected to positive selection (Antao et al., 2008). We identified *MC1R*1 loci in five goat populations as potential target of selection according to their genetic variation distribution by estimating the *F*_ST_ and mean heterozygosity as a measure of genetic differentiation at each locus, as six loci at five genes in goat populations were detected under positive selection by estimating their *F*_ST_ and heterozygosity (Pariset, Joost, Marsan, & Valentini, 2009). Earlier studies used microsatellite markers or AFLPs for selection detection, but recent studies using SNPs as genetic markers to identify genome wide selection signature due to their rapid discovery rate, as in humans and goat used SNPs as genetic markers to detect outlier loci subjected to positive selection (Pariset et al., 2009; Sunyaev, Lathe Iii, Ramensky, & Bork, 2000; Vallender & Lahn, 2004). The gene frequencies maintain polymorphism to equilibrium under balancing selection, whereas the directional selection reduces the genetic diversity within the population and among the population, as Kelley et al. (Kelley, Madeoy, Calhoun, Swanson, & Akey, 2006) found outlier approach determined positive selection for target genes. Hoffman, Schueler, Jones, & Blouin, (2006) showed that using Beaumont and Nichols (Beaumont & Nichols, 1996) approach, it is possible to find candidate gene under selection.

In an integrative approach to detect positive selection, all methods mapped codon evolution at various sites through SLAC, FEL, IFEL, and random effect likelihood and random effect likelihood methods in HyPhy package (Pond & Muse, 2005). REL detected five codons at sites 59, 218, 248, 327, and 364, while IFEL detected three codons at sites 59, 279, and 327 under positive selection (Table 2). Among these sites, 59 and 327 were detected as positively selected sites in both of the method and the sites 218, 248, and 364 were detected only in REL, it is because other approaches may lack the power of data set containing sequences with low divergence (Pond & Frost, 2005). SLAC and FEL analysis detected two sites under positive selection 59, 279 and 59, 364, respectively. The codon position 59 was identified under positive selection in all four analyses methods and which is strong evidence of positive selection of *MC1R* gene in goat. At position 59, the codon TCC in converted into TGC (C↔G) and the amino acid change on this site was serine (S) to cysteine (C). SLAC and FEL detected three and seven sites under positive selection for neuraminidase viruses, respectively (Li et al., 2011), as in another study, SLAC detected no site under selection, while FEL and REL detected two sites under positive selection and showed less false‐positive results (Sorhannus & Pond, 2006). In this study, we used various selection analysis methods to identify positive selection sites. We identified various codon sites under positive selection based on significance values (*p*‐value<.05) for each test and Bayes factor >20 for REL, as Kosakovsky et al. used SLAC, IFEL, REL, and FEL methods to detect positive selection in human HIV data based on test significance level (*p*‐value) and empirical Bayes factor for REL and identified various sites under positive selection at *p* < .05 and Bayes factor>20 (Pond et al., 2006). Our results contribute the discussion for Bayes factor‐based analysis and counting‐based method to detect positive selection. REL considered as preferable models to detect the codon sites under positive selection or negative selection (Suzuki, 2004; Suzuki & Nei, 2001).

The mixed‐effect model evolution approach based on empirical Bayes procedure identified diversifying selection at *MC1R* gene, as it estimated the LRT at a particular branch site and identified various sites under diversifying selection. MEME allows site to site and branch to branch distribution of ω and analyze the adaptive evolution at gene level (Yang & Dos Reis, 2011). In our study, we identified six codon sites in the *MC1R* gene under diversifying selection, and among these sites, there were two sites 59 and 364 (Table 3), which were identified under the selection in former analyses (REL, FEL, SLAC, and IFEL), which were strong evidence of adaptive evolution and selection at *MC1R* gene. In this analysis of diversifying selection, we restricted the analysis for only those sites with *p*‐value <.05 and empirical Bayes factor threshold > 20, and the best results are achieved when selected branches are placed in conserved lineage (Murrell et al., 2012). At codon site 59, MEME obtained maximum‐likelihood estimate β^+^ > α with *p* value .015, while, IFEL, SLAC, and FEL obtained maximum‐likelihood estimate β > α with *p* values .03, .02, and .02, respectively, and identified this site under positive selection. However, at codon 364, MEME obtained maximum‐likelihood estimate β^+^ > α with *p* value .03 and only REL identified 364 codon under positive selection based on empirical Bayes factor, but IFEL, SLAC, and FEL obtained maximum‐likelihood estimate β < α and *p*‐value >.05, which failed to infer positive selection. It was due to the fact that the natural selection is episodic with transient period of adaptive evolution masked by the prevalence of purifying selection or neutral selection and could not be identified under positive selection with less sensitive tests (Chen & Sun, 2011; Murrell et al., 2012). Inferring the distribution of gene‐specific selection parameters potentially improved to detect selection across many sites, and we further performed the FUBAR analysis to manifest the positive selection at *MC1R* gene. This study identified three sites (Table 4) under diversifying selection based on the conditional likelihood approach, and the codon sites (β > α) and empirical Bayes factor (EBF > 20) were identified under the diversifying selection (Murrell et al., 2013). These three sites were also identified under positive selection by former (REL, FEL, IFEL, SLAC) analyses, which are providing the strong evidence of diversifying selection at *MC1R* gene under selection lineage. Using MCMC convergence based on potential scale reduction and effective population size, we computed posterior probability of positive selection for each site and the positions under positive selection had PSRF values <1.03 and effective population size is more than 150 (Murrell et al., 2013).

The synonymous and nonsynonymous substitutions were identified by codon model substitution over nucleotide and amino acid substitutions (Miyazawa, 2011), and the rate variation significance over protein sites has been validated in nucleotide and amino acid substitution models (Yang, 1994). The synonymous and no‐synonymous substitution ratio was used to detect molecular footprints of *MC1R* gene based on amino acid residues exchange rates, as Huelsenbeck, Joyce, Lakner, & Ronquist, (2008) studied Bayesian approach to estimate empirical amino acid substitution models using the Dirichlet method to detect amino acids exchange rate classes. However, in our study rate class, three detected evolutionary finger prints of *MC1R* gene based on modified Bayesian Information Criterion (mBIC), which used 6680 models to estimate the amino acid exchangeability rate and the models with combined empirical codon and physicochemical parameters, such as transition/transversion rates, have shown evidence of selective effects with substitution preferences for particular amino acids (Kosiol, Holmes, & Goldman, 2007; Posada & Crandall, 1998). The genetic algorithm inferred three rate classes, three sites for *MC1R* alignment and observed the site to site amino acid variation to compute cluster cores. The genetic algorithm detected codon 59 under selection, as it has three rate classes (0.06, 0.19, and 0.45), and the transition ratio of amino acids was higher for this site. The best fit model with mBIC values 27,114.7 defined the cluster rate, and the numerical values of corresponding rate class substitution are inferred with maximum‐likelihood estimation, which are the computed evolutionary evidence ratio for the gene (Pond, Mannino, Gravenor, Muse, & Frost, 2007). We further performed the evolutionary fingerprint analysis based on probability distribution of site to site synonymous (α) and nonsynonymous (β) substitution rates in alignment and exploited the positive evolution of *MC1R* gene by estimating the ratio ω = β/α to manifest positive selection, as this ratio (ω = β/α) is an indicator of positive or negative selection in large adaptive evolutionary studies (Arbiza, Dopazo, & Dopazo, 2006; Nielsen et al., 2005).

The *MC1R* selection patterns discussed here reveal the utility of candidate gene approaches to adaptive phenotypic evolution that can be compared with methods based on genomic scans using neutral markers. Association studies using *MC1R* and other pigmentation loci may be valuable when studying both Mendelian and quantitative phenotypic traits, either on their own or in coincidence with neutral markers (Nachman, 2005). The drawbacks of candidate gene strategies are obvious when negative results are obtained; that is, there is no association between phenotype and genotype at the candidate locus. The negative inferences in such cases only narrate to the part of the gene studied; for example, in the Phylloscopus study stated by MacDougall‐Shackleton et al. (MacDougall‐Shackleton, Blanchard, & Gibbs, 2003), it is possible that there is an association with the small portion of *MC1R* coding region that was not sequenced or with the *MC1R* promoter region (Mundy, 2005). In general, the success of the approaches depends on various factors such as the accessibility of candidate genes; the evaluation of genetic variation viability in them and the number of loci involved in a particular trait evolution. Fortified selection of candidate genes rendering to the specificity of their phenotypic effects seems to be important, and deleterious effects at loci recognized in inbred captive lines may not always be expressed in captivity.

## 5 Conclusions

Collection of results from many selection analyses at *MC1R* gene provides an ideal opportunity to investigate adaptive selection that has influenced the coat color genetic variability and architecture of mammalian genome. As the strong selection of *MC1R* gene provides the comprehensive map of coat color genetic variability as well as pattern of selection at *MC1R* gene within the local goat breeds and its correlation among other animal species to reveal molecular evolution. Furthermore, this study will help to construct the data for understanding the selection signature for positive selection of gene among populations in animal breeding and allows the researchers to perform systematic approaches to detect the evolutionary footprints of selection at specific gene.

## Conflict of Interest

None declared.

## Authors’ Contributions

H.I. Ahmad wrote the original draft. H.I. Ahmad and H.H involved in the formal analysis. H.I Ahmad, Ch. Liu, and Y. C involved in methodology. Xp. J and G. Liu performed the supervision of the article.
