# Supplementary material for: Adaptive molecular evolution of MC1R gene reveals the evidence for positive diversifying selection in indigenous goat populations
Source: Ecol Evol. 2017 Jun 7;7(14):5170–80. doi: 10.1002/ece3.2919 (PMC5528238; doi:10.1002/ece3.2919)
Supplement: Supplementary file 1 [file ECE3-7-5170-s001.docx]

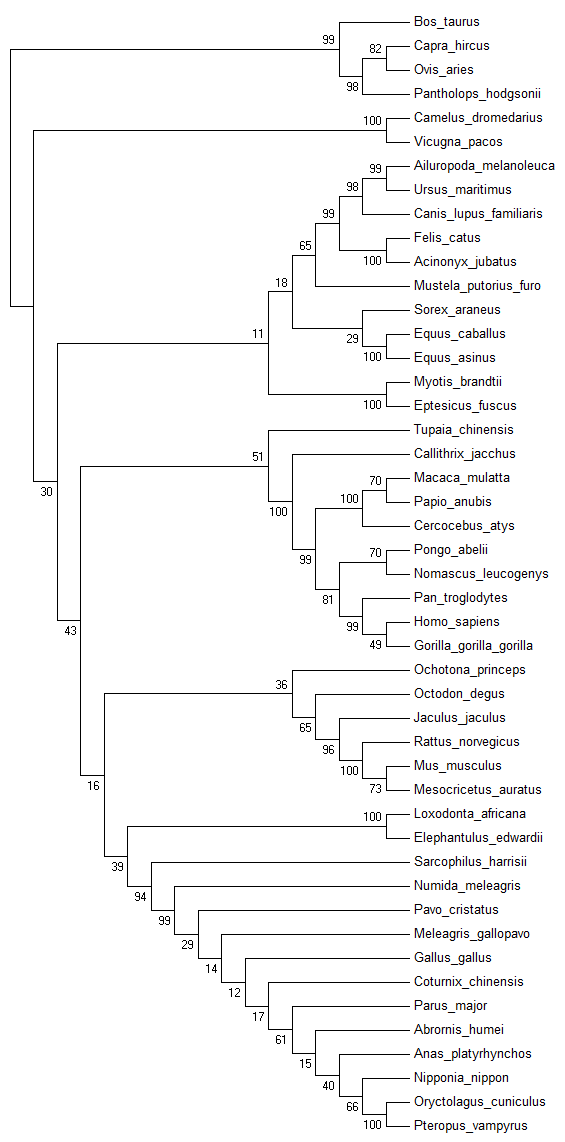


**Figure S1:** Phylogenetic relationship of *MC1R* gene was constructed. The taxa clustered together in the bootstrap test 1000 replicates are shown next to the branches based on Maximum Likelihood approach selecting the topology with higher log likelihood value and the branch length measured in the number of substitutions per site.
