# Supplementary material for: Adaptive molecular evolution of MC1R gene reveals the evidence for positive diversifying selection in indigenous goat populations
Source: Ecol Evol. 2017 Jun 7;7(14):5170–80. doi: 10.1002/ece3.2919 (PMC5528238; doi:10.1002/ece3.2919)
Supplement: Supplementary file 2 [file ECE3-7-5170-s002.docx]

**List of animal species of MC1R nucleotide sequences retrieved from GenBank**

| **Sr. No.** | **Species** | **GenBank Ref. No.** |
| --- | --- | --- |
| 1 | *Capra hircus* | XM_013970999.1 |
| 2 | *Ovis aries* | NM_001282528.1 |
| 3 | *Bos taurus* | NM_174108.2 |
| 4 | *Mus musculus* | NM_008559.2 |
| 5 | *Homo sapiens* | NM_002386.3 |
| 6 | *Rattus norvegicus* | XM_006255795.2 |
| 7 | *Cercocebus atys* | XM_012082356.1 |
| 8 | *Papio anubis* | NM_001164589.1 |
| 9 | *Pongo abelii* | XM_009251076.1 |
| 10 | *Ailuropoda melanoleuca* | XM_011234282.1 |
| 11 | *Canis lupus familiaris* | NM_001014282.2 |
| 12 | *Equus caballus* | NM_001114534.1 |
| 13 | *Acinonyx jubatus* | XM_015070588.1 |
| 14 | *Pteropus vampyrus* | XM_011379128.1 |
| 15 | *Camelus dromedarius* | XM_010998037.1 |
| 16 | *Loxodonta africana* | DQ648861.1 |
| 17 | *Elephantulus edwardii* | XM_006902856.1 |
| 18 | *Callithrix jacchus* | AY205120.1 |
| 19 | *Nomascus leucogenys* | AB296234.1 |
| 20 | *Sarcophilus harrisii* | XM_003759223.1 |
| 21 | *Ochotona princeps* | XM_012926641.1 |
| 22 | *Sorex araneus* | XM_004600791.1 |
| 23 | *Octodon degus* | XM_012517799.1 |
| 24 | *Jaculus jaculus* | *XM_004664824.1* |
| 25 | *Mustela putorius furo* | XM_013056885.1 |
| 26 | *Felis catus* | NM_001009324.1 |
| 27 | *Mesocricetus auratus* | XM_005073162.2 |
| 28 | *Oryctolagus cuniculus* | XM_008251954.1 |
| 29 | *Equus asinus* | XM_014836533.1 |
| 30 | *Myotis brandtii* | XM_014535606.1 |
| 31 | *Pantholops hodgsonii* | FJ773348.1 |
| 32 | *Tupaia chinensis* | XM_006152522.2 |
| 33 | *Macaca mulatta* | XM_015126842.1 |
| 34 | *Pan troglodytes* | NM_001009152.1 |
| 35 | *Gorilla gorilla gorilla* | XM_004058178.1 |
| 36 | *Vicugna pacos* | FJ502229.1 |
| 37 | *Eptesicus fuscus* | XM_008140011.1 |
| 38 | *Ursus maritimus* | XM_008702716.1 |
| 39 | *Pavo cristatus* | KF379748.1 |
| 40 | *Gallus gallus* | NM_001031462.1 |
| 41 | *Meleagris gallopavo* | GU905059.1 |
| 42 | *Anas platyrhynchos* | NM_001310805.1 |
| 43 | *Coturnix chinensis* | KC253402.1 |
| 44 | *Nipponia nippon* | KC190164.1 |
| 45 | *Numida meleagris* | GQ449249.1 |
| 46 | *Abrornis humei* | AY308750.1 |
| 47 | *Parus major* | XM_015639517.1 |

**Table: List of Primers used in study**

| **Gene** | **Variant** | **Primer(*5'→3'*)** | **Annealing** |  |  |
| --- | --- | --- | --- | --- | --- |
| Agouti | G/A | F:AAACCGTGCAGCTGAGTGGGATCCCGC | 55℃ |  |  |
|  |  | R:TGTCCACAGAACACCCTA |  |  |  |
| GnRHR | -/G | F:TCTTGAAGCTGTATCAGCCATA | 59.3℃ |  |  |
|  |  | R:GTGTTGAAAATTGTGGAGAGTAGA |  |  |  |
| INH | T/C | F:ATACGGATTGCCTGTG | 54℃ |  |  |
|  |  | R:ACGCCAACTACTGTGAG |  |  |  |
| FSHβ | G/T | F:TTGGCTTCTGTTACATCTC | 54.5℃ |  |  |
|  |  | R:CTTTCCTGAAATGTCCTTGC |  |  |  |
| PGR | G/A | F:ATGACTGAGCTGAAGGCGA | 65℃ |  |  |
|  |  | R:CGTCTGCCAGCGACTCTG |  |  |  |
| Myf-5 | A/C | F:TGGACATGATGGACGGCT | 61.5℃ |  |  |
|  |  | R:CATGCCATCAGAGCAACTTG |  |  |  |
| NPY-YR | C/G | F:GGTCTCTGGAAACCTGGCTTTGAT | 58℃ |  |  |
|  |  | R:TTGGTCTCCAACCCCGAGGATTGAT |  |  |  |
| IGFα | G/C | F:CACAGCGTATTATCCCAC | 55℃ |  |  |
|  |  | R:GACACTATGAGCCAGAAG |  |  |  |
| AA-NAT | C/T | F:ATGTCCACGCCGAGCATCCACT | 59℃ |  |  |
|  |  | R:CCTCTCGCTCAATCTCAAACACG |  |  |  |
| LHPα | C/A | F:ACCAGATCTTGGCCCTTG | 57℃ |  |  |
|  |  | R:CAAAGCCTGAGTCCAAC |  |  |  |
| LHPβ | T/C | F:CCTGAGGCACTGGCCTGTCC | 66℃ |  |  |
|  |  | R:CACCATGCTGGGGCAGTAGCC |  |  |  |
| MC1Rα | G/T | F:CCATCCCTGACGGGCTCTTT | 64℃ |  |  |
|  |  | R:GGGAGTGCAGGTTGCGGTTC |  |  |  |
| MC1Rβ | G/A | F: ACACGGTCGTCCTGCTGTGC | 62℃ |  |  |
|  |  | R: AGCGCCCTTGAGGCCAAAGC |  |  |  |
